# What could infant and young child nutrition learn from sweatshops?

**DOI:** 10.1186/1471-2458-11-276

**Published:** 2011-05-05

**Authors:** Peter A Singer, Sean Ansett, Isabella Sagoe-Moses

**Affiliations:** 1 McLaughlin-Rotman Centre for Global Health, University Health Network & University of Toronto, 101 College Street, Suite 406, Toronto, Ontario, M5G 1L7, Canada; 2 Sean Ansett, Masters of Studies Sustainability Leadership, Candidate, University of Cambridge, Wolfson College, UK; 3 National Child Health Coordinator; Reproductive & Child Health Unit, Ghana Health Service, Private Mail Bag, Ministries-Accra, Ghana

## Abstract

**Background:**

Adequate infant and young child nutrition demands high rates of breastfeeding and good access to nutrient rich complementary foods, requiring public sector action to promote breastfeeding and home based complementary feeding, and private sector action to refrain from undermining breastfeeding and to provide affordable, nutrient rich complementary foods. Unfortunately, due to a lack of trust, the public and private sectors, from both the North and the South, do not work well together in achieving optimal infant and young child nutrition.

**Discussion:**

As the current debate in infant and young child nutrition is reminiscent of the "sweatshop" debate fifteen years ago, we argue that lessons from the sweatshops debate regarding cooperation between public and private sectors - and specific organizational experiences such as the Ethical Trading Initiative in which companies, trade unions, and civil society organizations work together to enhance implementation of labour standards and address alleged allegations - could serve as a model for improving cooperation and trust between public, civil society and private groups, and ultimately health, in infant and young child nutrition.

**Summary:**

Lessons from the sweatshops debate could serve as a model to promote cooperation and trust between public and private groups, such that they learn to work together towards their common goal of improving infant and young child nutrition.

## Background

Eight million children die each year before their fifth birthday - and malnutrition plays an important role in one third of these deaths. Moreover, the period between conception and two years of age is a particularly vulnerable time when under-nutrition and micronutrient deficiencies lock an entire generation into diminished physical and intellectual development, undermining human capital in the developing world. The health Millennium Development Goals will not be achieved without action to reduce malnutrition in infants and young children [1].

The World Health Organization (WHO) recommends that infants be exclusively breastfed for the first six months of life. Thereafter, to meet their evolving nutritional requirements, infants should receive nutritious and safe solid foods which complement, but not substitute, breastfeeding through the first two years or beyond [1]. These complementary foods enrich the diet with a broad range of micronutrients (including Vitamin A, iron, zinc), essential fats, and energy. Therefore, adequate Infant and Young Child Nutrition (IYCN) demands high rates of breastfeeding and good access to nutrient rich complementary foods, requiring public sector action to promote breastfeeding and home based complementary feeding, and private sector action to refrain from undermining breastfeeding and to provide affordable, nutrient rich complementary foods.

Unfortunately, the public and private sectors do not work well together to achieve optimal IYCN. Past, and in some cases ongoing, misbehaviours on the part of companies, dating back to concerns about the promotion of infant formula leading to the 1977 boycott of Nestlé, have led to acrimony, distrust, and a lack of coordinated efforts. The authors attended a workshop in the summer of 2009 that included a range of public and private stakeholders and were surprised to learn that the participants could not recall a time when public and private groups engaged with infant and young child nutrition had sat in the same room to share views and concerns. Yet, improved cooperation between public, civil society and private groups, from both the North and the South, in infant and young child nutrition could build trust, and lead to saving and improving the lives of children.

The current state of affairs in IYCN is reminiscent of the "anti-sweatshop" debate fifteen years ago - recall the criticism of Nike with respect to the use of child labour in the manufacture of soccer balls in Pakistan [2] - where there was also mistrust and little collaboration between public and private sectors. Thanks to risktaking by some stakeholders, including trade unions, advocacy organizations and companies, there is today more collaboration between the public and private sectors with respect to the enforcement of child labour standards, including informal and formal complaint mechanisms to manage issues collaboratively when violations of working conditions occur.

In this article, we argue that lessons from the sweatshops debate regarding cooperation between public and private sectors - and specific organizational experiences such as the Ethical Trading Initiative in which companies, trade unions, and civil society organizations work together to enhance implementation of labour standards and address alleged violations - could serve as a model for improving trust, and ultimately health, in infant and young child nutrition. Our discussion will focus on the developing world where mistrust between these sectors is highest; however, the lessons we present could also be applied to infant and young child nutrition in the developed world.

## Discussion

### The International Code of Marketing of Breast-milk Substitutes

The International Code of Marketing of Breast-milk Substitutes (the Code) was developed twenty-nine years ago to ensure that aggressive marketing practices used by companies selling infant formula are regulated, and that these practices do not undermine the critical role breast-milk plays in optimal infant and young child nutrition [3]. Its scope includes all breast-milk substitutes when marketed as a partial or total replacement for breast-milk, and the Code outlines acceptable marketing including:

• No gifts or samples to health care workers.

• No free or low cost supplies to health care system.

• No free samples to mothers or their families.

• No promotion of products, which include product displays, posters or distribution of promotional materials.

• No deployment by companies of nurses or other company paid personnel to provide postnatal care.

• Technical product information can be given to health workers but this must be factual and scientific.

• Informational and educational materials should explain the benefits and superiority of breastfeeding, the health hazards associated with bottle feeding, and the costs of using infant formula [3].

The Code has helped to raise awareness about the importance of breastfeeding and its role in infant and young child nutrition, put the issue on the agenda of global institutions, and helped governments adopt national policies. For example, Ghana's Breastfeeding Promotion Regulations 2000 legislation was a long fought attempt to raise awareness about breastfeeding; 63% of Ghanian children below 6 months are now exclusively breastfed [4].

But from a global standpoint progress has been modest. Between 1995 and 2008, exclusive breastfeeding on infants between 0 and 6 months of age in the developing world increased from 33% to only 37% [5]. The global baby foods and infant formula market is projected to grow steadily, reaching US $20.2 billion in sales by 2010, especially in more lucrative and populous developing world markets such as India and China [6]. According to NGO International Baby Food Action Network, whose reporting is supported by UNICEF and WHO, not one food company that markets infant formula fully complies with the Code [7].

NGOs claim that companies deliberately violate the code by advertising their product inappropriately, capitalizing on the weak monitoring systems and inadequate awareness that exists even in some countries with legislation related to the Code, including under resourced and corrupt governments. For example, according to NGO Baby Milk Action in the Argentina case: "... Nestlé hides behind its bogus interpretation of the World Health Assembly's marketing requirements to continue with business as usual, putting its own profits before infant health" [8]. Public sector organizations are sometimes reluctant even to engage in dialogue with companies, possibly out of concern that engagement might be misinterpreted as endorsement; that constituencies will conclude that they are privately profiting in some way from the relationship; or that the risks of engaging outweigh any perceived benefits of the private sector's contribution to improving the nutrition of the world's impoverished infants and young children.

Corporate stakeholders argue that in developed countries women have entered the workforce and should be able to choose how they feed their babies and the risks of bottle feeding are diminished with reliable access to clean water. Moreover, the companies claim the Code has shortcomings: it is open to interpretation and is unclear with respect to its definition of complementary foods; it is difficult for a company in violation of the Code to come out of this status; and those charged with monitoring the Code's implementation at times have presented evidence that is flawed. For example, in 2003 the NGO Baby Milk Action and Nestle disagreed about whether a whole milk marketed in Argentina was in fact a breast-milk substitute, with Nestle claiming that "Baby Milk Action (BMA) confuses whole-milk with infant formula" [8].

A recent working paper from the Global Alliance for Improved Nutrition has aimed to clarify the application of the Code to commercially available complementary foods [9]. Nevertheless, private sector firms are reluctant to enter developing world markets for complementary foods because they worry they will be attacked for undermining breastfeeding, and this will expose them to unwanted publicity and hurt their brand, as well as potentially their license to operate, in established markets.

An astute commentator has recently noted: "The controversy that has bedeviled the code for 30 years is almost entirely limited to matters of interpretation and compliance. However, because these issues have been so protracted it has led to an atmosphere of mistrust that has now become embedded ... It is, therefore, timely to reflect on the vision of those who initiated the code and to embrace the original spirit of the code which is that participation and cooperation are essential if activities aimed at the improvement of maternal, infant and child nutrition are to be successful. To enable this to happen, it is proposed that measures are taken to replace current hostilities with effective national and international governance." [10]

### Anti-sweatshop movement

The current debate in infant and young child nutrition is reminiscent of the "sweatshop" debate fifteen years ago. At that time, campaigning, protests, and polarization between stakeholders was the norm and little progress had been made on actually improving working conditions in global supply chains due to a lack of dialogue and mistrust between the parties involved. In the "sweatshop" debate, companies, civil society and trade unions had different views on issues like trade liberalization and "living wage." Despite these differences, which still exist, stakeholders were able to define their common area of interest - to improve implementation of a code of conduct of labour practices.

Concrete evidence of this collaborative approach was the emergence of multistakeholder initiatives (MSIs) - such as the Ethical Trading initiative in the UK, Social Accountability International in New York, and the Fair Labor Association in Washington, DC - as an attempt to see if these parties could work together on joint solutions to complex labour issues in global supply chains. MSIs are "characterized by multi-stakeholder governance structures and activities, and by mechanisms for enforcement through mutual accountability, market leverage, and/or non-market pressures (both regulatory and non-regulatory)." [11] They incorporate multiple sectors in their decision making processes: in the case of the Ethical Trading Initiative the governance structure includes companies, NGOs, and Trade Unions who have equal voting rights and are mutually accountable for the decisions they take and the overall strategy of the organization. Because collaboration is a necessary requirement of effectiveness, partners in an MSI must be able to hold each other to a set of standards, to judge whether they have fulfilled their responsibilities in light of these standards, and to impose sanctions if they determine that these responsibilities have not been met [12]. Although including diverse stakeholders can slow down decision making, since consensus needs to be agreed from sectors that typically have very different views and approaches, once the sectors are brought in they can leverage each others' networks, strengths, and experiences. Additionally, MSIs benefit from improved credibility and transparency since the initiative has accounted for key stakeholder views.

While campaigning was effective at raising awareness about sweatshop conditions, campaigning alone had limitations because trying to tackle labour issues in a global economy where a multinational may be producing in thousands of factories in more than 50 different countries proved difficult. Moreover, much of the production was taking place in developing countries where enforcement mechanisms may be weak. It took years for the relevant stakeholders (companies, trade unions, governments) to realize that no one sector alone could resolve sweatshop related issues. Companies like Levi's, Gap, and Nike were playing a key role in developing solutions. Ultimately, engagement between unlikely partners has had positive effects on child labour and health and safety issues.

A concrete example of a collaborative approach in the sweatshops debate that could serve as a model for infant and young child nutrition is the UK based Ethical Trading Initiative (ETI), a voluntary alliance of companies (with over 65 firms including the Body Shop, Marks and Spencer's, and Tesco), trade unions, and NGOs concerned with labour standards. Their code of standards is based upon International Labour Organization's conventions. When allegations of violations are raised, the complaint can be brought to the company or to ETI itself. If the complaint is not remedied, stakeholders within ETI can call for a commission to further investigate and arbitrate the complaint.

ETI has had impact in developing countries and the UK. In South Africa, two farms no longer employ children on school holidays; in one case the supplier had assisted the children with school fees [13]. Suppliers in India and Vietnam now provide regular workers with legal entitlements such as Employees State Insurance and Provident Fund (India), and annual and maternity leave (Vietnam). In Costa Rica, working hours are being monitored at three sites to ensure packhouse and harvest workers do not exceed the 60 hour limit. In the UK, lobbying efforts have led to adoption of legislation on gang masters (individuals who took advantage of migrant workers in the agricultural sector in the UK) and a homeworker's minimum wage that was passed by the UK government.

### From sweatshops to infant nutrition

Imagine an Ethical Trading Initiative for infant and young child nutrition. Companies, government, and civil society organizations would work together to monitor code implementation and address alleged violations. These public and private stakeholders would share their perspectives and learn to work together towards their common goal of improving infant and young child nutrition.

A good starting point for collaboration would be agreement on the part of stakeholders that early initiation and exclusive breastfeeding for the first six months, and the timely introduction and appropriate use of complementary foods, is the best way to improve infant and young child nutrition. Such a holistic view puts the emphasis on optimal infant and young child nutrition, rather than pitting one modality (breastfeeding or complementary feeding) against the other when both are essential. All parties could work toward this holistic goal.

The ETI model could be applied by initially piloting one developing country. Code experts agree that the ultimate goal should be for national governments to develop platforms that support Code compliance, and that effective national implementation of the Code requires capacity to monitor compliance with and enforce the legislative framework [14]. The capacity to monitor and enforce the Code at the national level could be provided by a national ETI-like organization. There is a critical need to bring the Code stakeholders together in a transparent and open manner to tease out differences and commonalities, manage complaints at the national level, provide remediation and remedies in real time in order to ensure the timely resolution of violations, and encourage open and transparent debate about the violations themselves.

Infant nutrition seems to have fallen into the trap of the 'Tyranny of Principles' identified by philosopher Stephen Toulmin - the inability of groups to agree on abstract principles, even when they can reach rapid agreement on particular cases. An ETI-like process would shift the focus from principles to cases where stakeholders would be more likely to find common ground.

Other approaches such as a shared code of principles for infant and young child nutrition [15], a forthcoming 'access to food' transparency index for food companies being developed by the Global Alliance for Improved Nutrition, and social auditing of potentially controversial projects [16] could also contribute to building trust among public and private stakeholders and reinforce these national efforts.

We acknowledge that our application of the "sweatshop debate model" to improve infant and young child nutrition has some limitations. Sweatshops-related child labour and the decline in breastfeeding rates are different social phenomena driven and influenced by diverse and distinct factors. While promoting co-operation and trust between the private and public sectors will be one essential component to improving infant and young child nutrition, this will not necessarily have an enduring influence on the maternal decision to breastfeed or not. Moreover, the sweatshop debate has taken more than a century to bring about significant results. It may, therefore, also take time for the stakeholders in infant and young child nutrition to find common ground and work together.

## Conclusions

Optimal infant nutrition plays a vital role in creating human capital, building strong communities, reducing poverty, growing economies and making countries more competitive over the long term. Improving the nutrition of populations is in the interest of companies, civil society and governments. Imagine a world where these actors, rather than fighting with one another, collaborate and leverage their skills to improve infant and young child nutrition. The field of infant and young child nutrition has much to learn from sweatshops.

## Competing interests

PAS has been a consultant for Pepsico Inc on a topic unrelated to this article. SA has been a consultant to the Global Alliance for Improved Nutrition on this topic. ISM has worked, on a volunteer basis, for IBFAN and its local network, the Ghana Infant Nutrition Action Network.

## Authors' contributions

All the authors discussed related issues together with others in a one day session that was the genesis of the idea. PAS wrote the first draft based on materials provided by SA. All authors reviewed that draft for critical content. All authors read and approved the final manuscript.

## Pre-publication history

The pre-publication history for this paper can be accessed here:

http://www.biomedcentral.com/1471-2458/11/276/prepub
